# Cheminformatics-Based Drug Design Approach for Identification of Inhibitors Targeting the Characteristic Residues of MMP-13 Hemopexin Domain

**DOI:** 10.1371/journal.pone.0012494

**Published:** 2010-08-31

**Authors:** Roopa Kothapalli, Asif M. Khan, Basappa, Anupriya Gopalsamy, Yap Seng Chong, Loganath Annamalai

**Affiliations:** 1 Department of Obstetrics and Gynaecology, Yong Loo Lin School of Medicine, National University of Singapore, Singapore, Singapore; 2 Department of Biochemistry, Yong Loo Lin School of Medicine, National University of Singapore, Singapore, Singapore; 3 Singapore-MIT Alliance for Research and Technology, Centre for Life Sciences, Singapore, Singapore; Aarhus University, Denmark

## Abstract

**Background:**

MMP-13, a zinc dependent protease which catalyses the cleavage of type II collagen, is expressed in osteoarthritis (OA) and rheumatoid arthritis (RA) patients, but not in normal adult tissues. Therefore, the protease has been intensively studied as a target for the inhibition of progression of OA and RA. Recent reports suggest that selective inhibition of MMP-13 may be achieved by targeting the hemopexin (Hpx) domain of the protease, which is critical for substrate specificity. In this study, we applied a cheminformatics-based drug design approach for the identification and characterization of inhibitors targeting the amino acid residues characteristic to Hpx domain of MMP-13; these inhibitors may potentially be employed in the treatment of OA and RA.

**Methodology/Principal Findings:**

Sequence-based mutual information analysis revealed five characteristic (completely conserved and unique), putative functional residues of the Hpx domain of MMP-13 (these residues hereafter are referred to as HCR-13_pf_). Binding of a ligand to as many of the HCR-13_pf_ is postulated to result in an increased selective inhibition of the Hpx domain of MMP-13. Through the *in silico* structure-based high-throughput virtual screening (HTVS) method of Glide, against a large public library of 16908 molecules from Maybridge, PubChem and Binding, we identified 25 ligands that interact with at least one of the HCR-13_pf_. Assessment of cross-reactivity of the 25 ligands with MMP-1 and MMP-8, members of the collagenase family as MMP-13, returned seven lead molecules that did not bind to any one of the putative functional residues of Hpx domain of MMP-1 and any of the catalytic active site residues of MMP-1 and -8, suggesting that the ligands are not likely to interact with the functional or catalytic residues of other MMPs. Further, *in silico* analysis of physicochemical and pharmacokinetic parameters based on Lipinski's rule of five and ADMET (absorption, distribution, metabolism, excretion and toxicity) respectively, suggested potential utility of the compounds as drug leads.

**Conclusions/Significance:**

We have identified seven distinct drug-like molecules binding to the HCR-13_pf_ of MMP-13 with no observable cross-reactivity to MMP-1 and MMP-8. These molecules are potential selective inhibitors of MMP-13 that can be experimentally validated and their backbone structural scaffold could serve as building blocks in designing drug-like molecules for OA, RA and other inflammatory disorders. The systematic cheminformatics-based drug design approach applied herein can be used for rational search of other public/commercial combinatorial libraries for more potent molecules, capable of selectively inhibiting the collagenolytic activity of MMP-13.

## Introduction

MMP-13 (Collagenase 3) is a zinc dependent protease which catalyses the cleavage of type II collagen, the main structural component of articular cartilage [1]. It is capable of cleaving the peptide bond at amino acid positions 775–776 in all three strands of the mature triple helical type II collagen molecules [2]. MMP-13 is expressed in articular cartilage and joints of osteoarthritis (OA) and rheumatoid arthritis (RA) patients, respectively, but not in normal adult tissues [3], [4].

Preclinical data implicate human MMP-13 as the direct cause of irreversible cartilage damage in arthritic conditions [4], [5], [6], [7]. This is supported by the findings that i) over expression of MMP-13 induces OA in transgenic mice, ii) its mRNA expression co-distributes with type II collagenase activity in osteoarthritic cartilage, and iii) an inhibitor of MMP-13 has been shown to disrupt the degradation of explanted human osteoarthritic cartilage. In arthritic syndromes, the expression of MMP-13 is elevated in response to the inflammatory signals by leukocytes and other immune cells, in particular interleukin 1 (IL-1) and tumour necrosis factor alpha (TNF-α) [3]. The increased levels of MMP-13 result in imbalance in their regulation by tissue inhibitors of metalloproteinases (TIMPs), thus likely contributing to the diseased state [8].

As a result, the MMP-13 protease has been a target for the inhibition of the progression of OA and RA. Early broad spectrum MMP inhibitors directed towards the zinc region of the catalytic domain (inhibitors exploiting the hydroxamate function as a zinc-binding group) have been ineffective because of their dose limiting toxicity in the form of musculoskeletal syndrome (MSS), characterised by joint stiffness and inflammation [9]. Conversely, specific inhibitors targeting the non-zinc region of the catalytic domain have been shown to effectively reduce the cartilage damage [4]. Recent studies have, therefore, focused on the search for selective inhibitors of MMP-13 [9], [10], [11]. The Hpx domain of the protease [12], [13], [14], which is critical for substrate specificity, represents an alternative target for the search of such inhibitors.

All MMPs in general have similar domain architecture, namely an N-terminal signal sequence to target for secretion, a pro-peptide domain to maintain latency for cell signalling, a catalytic domain containing catalytic zinc binding motif, a linker region that links the catalytic domain region with the C-terminal four bladed propeller structure Hpx domain [15]. The catalytic domain of these MMPs are unable to cleave the triple helical collagens without the Hpx domain [16]. Further, the removal of the Hpx domain from MMP-1, -8 and -13, which belong to the collagenase family, has been shown to result in a loss of collagenolytic activity [13]. Thus, the Hpx domain in the C-terminal region maintains the specificity of collagenase family MMPs by affecting the substrate binding [2].

In this study, we applied a cheminformatics-based drug design approach to i) define the putative characteristic functional residues of the Hpx domain of MMP-13, ii) identify and characterize ligands binding to these residues and iii) assess the selectivity of these ligands by testing their cross-reactivity to other collagenase family members, MMP-1 and -8. Such screened and selected potential specific inhibitors can then be tested by molecular experiments to validate their specificity to MMP-13 and their application as drug targets.

## Materials and Methods

### Sequence-based analysis to identify putative characteristic functional residues of the Hpx domain of MMP-13

The identity of characteristic residues specific to the Hpx domain of MMP-13 have not been reported previously [13]. We conducted sequence-based analyses to identify these amino acid residues by performing a multiple sequence alignment and using the AVANA tool (http://sourceforge.net/projects/avana/) to compare the mutual information between subsets of the alignment for the location of the characteristic sites [17].

The sequences of all reported human MMP proteins were retrieved by performing PSI-BLAST [18] search against the non-redundant (nr) NCBI Entrez protein database using the MMP-13 query sequence obtained from the Protein Data Bank [19] (PDB ID:1PEX). A total of 50 MMP sequences were obtained from the BLAST search (Table S1 and Table S2). These sequences were then aligned using Muscle v3.6 [20] and the resulting alignment was manually inspected and corrected for misalignments using BioEdit [21]. The regions of the alignment representing the pro-peptide domain, catalytic domain and the linker region were deleted, leaving only the Hpx domain.

The alignment of the MMP Hpx domain sequences was then submitted to AVANA to identify residues that are completely conserved and characteristic to MMP-13 (*i.e.* characteristic residues are defined as those with 100% amino acid identity and mutual information value of 1). AVANA has a built-in functionality to identify conserved, characteristic sites between subsets of sequences in an alignment using entropy and mutual information theories [17]. Herein, the two subsets for our alignment in AVANA were i) 8 MMP-13 sequences and ii) all other MMPs (42 of them). Having identified the Hpx characteristic residues (abbreviated as HCR for brevity) of MMP-13 (i.e. HCR-13), those that matched the putative functional residues of Hpx [15] were identified (abbreviated as HCR-13_pf_).

Two main caveats herein include the small sample size and the sampling bias for the MMP sequences reported in the public database. However, the data used in this study was the most representative and comprehensive available in the public database to date (May 2009). Further, the characteristic residue list can be refined with the availability of more sequence data in the future.

### Virtual screening

We next aimed to identify and characterize ligands that interact with the HCR-13_pf_. The *in silico* structure-based high-throughput virtual screening (HTVS) method of Glide, version 5.5 (Schrödinger, LLC, New York, 2009) [22], was used to identify potential ligand molecules that interact with at least one of the HCR-13_pf_ residues on the 3D structure of MMP-13 (PDB ID: 1PEX). The binding of ligands to these residues is postulated to render selectivity to the inhibition of the proteolytic activity of the enzyme MMP-13. A total of 16908 molecules derived from public libraries namely Maybridge (14400; www.maybridge.com), PubChem [23] (2438; obtained from Shanghai Institute of Organic Chemistry) and Binding (70; www.bindingdb.org), were selected for virtual screening against 1PEX.

Before performing HTVS, hydrogen atoms and charges were added to the crystal structure of 1PEX and then the complex was submitted to a series of restrained, partial minimizations using the optimized potentials for liquid simulations all-atom (OPLS-AA) force field [24]. The 3D structure was processed by use of the ‘Protein Preparation module’ with the ‘preparation and refinement’ option before docking. The grid-enclosing box was centred to all HCR-13 residues in 1PEX, so as to enclose the residues within 3 Å from their centroid. A scaling factor of 1.0 was set to van der Waals (VDW) radii for these residue atoms, with the partial atomic charge less than 0.25. The ligand molecules collected from the databases were prepared using ‘LigPrep’ module and were subsequently subjected to Glide ‘Ligand docking’ protocol with HTVS mode.

### Glide extra precision docking for the screened ligands

All the ligands selected from the screening step were then subjected to Glide docking with extra precision (XP) to identify residues involved in hydrogen bond interactions with 1PEX. Glide XP mode determines all reasonable conformations for each low-energy conformer in the designated binding site. In the process, torsional degrees of each ligand are relaxed, though the protein conformation is fixed. During the docking process, the Glide scoring function (G-score) was used to select the best conformation for each ligand. Final G-scores were selected based on the conformation at which the identified ligands formed hydrogen bonds to at least one of the HCR-13_pf_ with optimal binding affinity. The docking procedures were performed on a Dell RHEL 5.0 workstation.

The ligands were then assessed for cross-reactive binding to MMP-1 and -8, using Glide XP; these MMPs were analysed because they also contribute to collagenolytic activity and contain an Hpx domain as MMP-13. The better resolution 3D structure for MMP-1 (1SU3 with catalytic and Hpx domains) and -8 (1BZS, only containing catalytic domain; no structure available with Hpx domain) obtained from PDB were used for the docking. The binding analysis on these structures was focused on the known active site residues of the catalytic domain of MMP-1 [25] and -8 [26] and the reported putative functional residues of Hpx domain of MMP-1 (285–295; Asp-Ala-Ile-Thr-Thr-Ile-Arg-Gly-Glu-Val-Met) [13]. It is noted that when aligned, the positions of the reported putative functional residues of the Hpx domain of MMP-1 do not correspond to those reported for MMP-13. This may be because of the selectivity of these two MMPs to different substrates, such as type I collagen for MMP-1 and type II for MMP-13 [27].

### Assessment of drug-like properties of selected optimized ligands

The selected optimized lead molecules from the cross-reactivity assay were studied for their drug-like properties based on Lipinski's rule of five [28], by use of the ADME-Tox application at the Mobyle portal (http://mobyle.rpbs.univ-paris-diderot.fr). The percentage of their human oral absorption was also predicted to determine the toxicity levels, by use of QikProp version 3.2, Schrödinger, LLC, New York, NY, 2009 [29].

## Results and Discussion

**Figure 1:**
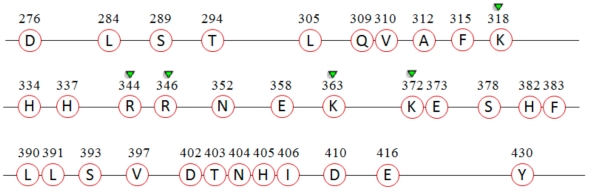
The characteristic residues of the Hpx domain of MMP-13 (HCR-13). The residues potentially important for the function of the domain (HCR-13_pf_) are indicated with the green inverted triangles. The amino acid positions are with respect to the Hpx domain of the PDB record 1PEX.

In this study, we identified 34 characteristic residues for the Hpx domain of MMP-13 (HCR-13) that were completely conserved and unique to the analyzed sequences of this domain (Figure 1). Five (Lys318, Arg344, Arg346, Lys363 and Lys372) of these were part of the 11 putative functional residues of Hpx [15] (these five are referred to as HCR-13_pf_). Binding of a ligand to as many of these HCR-13_pf_ and possibly the remaining HCR-13 are postulated to result in increased selective inhibition of the Hpx domain of MMP-13. Through HTVS, we identified 25 ligands that interact with at least one of the HCR-13_pf_. The ligands were screened from a large library of 16908 molecules obtained from the public databases Maybridge, PubChem and Binding; all the identified 25 ligands were from Maybridge.

Docking analysis using the more precise XP mode of Glide revealed that the 25 ligands formed hydrogen bonds with 1–3 residues of HCR-13, of which 1–2 were HCR-13_pf_. In addition, hydrogen bonds were also formed by 1–2 non-HCR-13 putative functional residues and 1 non-HCR-13 non-putative functional residue (Table S3).

Assessment of cross-reactivity of the 25 ligands with MMP-1 (containing both catalytic and Hpx domains) and MMP-8 (only catalytic domain), members of the collagenase family as MMP-13, returned seven lead molecules that did not bind to any one of the putative functional residues of Hpx domain of MMP-1 and any of the catalytic active site residues of MMP-1 and -8. Also, the closest distance between the putative functional residues of the Hpx (MMP-1) or the catalytic active site residues (MMP-1 and MMP-8) to the lead molecules was more than 10 Å (data not shown), suggesting that the ligands are not likely to interact with the functional or catalytic residues. The docking results of the final seven lead molecules to 1PEX are given in Table 1.

**Table 1:** Glide extra-precision (XP) results for the seven lead molecules, by use of Schrodinger 9.0.

Lead molecules a	G-score (kcal/mol) b	Interacting amino acids (HBD Å) c	#HB d	Type of Interaction e
3764	−9.22	**ARG344** (1.491), **ARG346** (1.963), LYS347 (1.869), *ASN326* (2.258)	4	polar
764	−9.07	**ARG344** (2.037), ARG300 (1.689 and 1.821)	3	polar
13196	−8.78	**ARG344** (1.748), ARG300 (1.595 and 1.853)	3	polar
3705	−8.74	**ARG344** (1.833 and 1.967), LYS347 (2.412), *ASN326* (1.903)	4	polar
632	−8.08	**ARG344** (1.923 and 1.877), *ARG326* (1.830)	3	polar
7789	−7.59	**ARG344** (2.324), **ARG346** (1.929), LYS347 (1.964 and 2.342)	4	polar
1598	−7.55	**ARG344** (1.468), *ASN326* (2.048)	2	polar

a Ligand IDs are of the Maybridge database.

b Glide score.

c The amino acids of the HCR-13_pf_ that interact with the lead molecules are in boldface and underlined, while functionally important residues of MMP-13 that are not part of HCR-13 are only underlined. Residues not functionally defined and not part of HCR-13 are in italics. The hydrogen bond distances, in angstrom (Å), between the interacting amino acids of 1PEX and the seven lead molecules are indicated in brackets.

d Number of hydrogen bonds formed.

e The amino acids exhibited polar contacts with the seven lead molecules.

**Figure 2:**
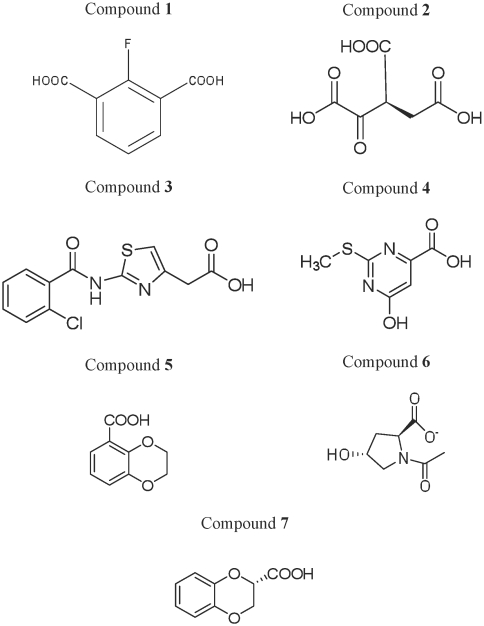
Structure of the seven lead molecules. The Maybridge database ID of the lead molecules are as follows: compound 1–3764; compound 2–764; compound 3–13196; compound 4–3705; compound 5–632; compound 6–7789; and compound 7–1598.

The chemical name of the seven lead compounds with their corresponding Maybridge identity (ID) number are 2-fluoroisophthalic acid (compound **1: 3764**), 3-(carboxymethyl)-2-methylenepentanedioic acid (compound **2: 764**), 2-{2-[(2-chlorobenzoyl)amino]-1,3-thiazol-4-yl}acetic acid (compound **3: 13196**), 6-hydroxy-2-(methylsulfanyl)-4-pyrimidinecarboxylic acid (compound**4: 3705**), 2,3-dihydro-1,4-benzodioxine-5-carboxylic acid (compound **5: 632**), 1-acetyl-4-hydroxypyrrolidine-2-carboxylic acid (compound **6: 7789**) and 2,3-dihydro-1,4-benzodioxine-2-carboxylic acid (compound **7: 1598**). The chemical structures of these lead molecules are illustrated in Figure 2.

**Figure 3:**
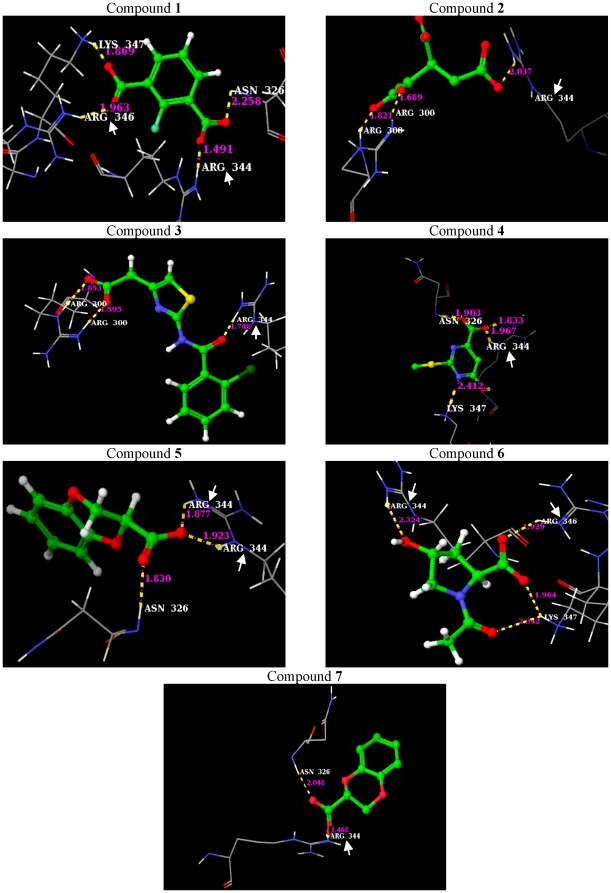
Binding poses of the seven lead molecules. The proposed binding mode of the lead molecules are shown in ball and stick display and non carbon atoms are coloured by atom types. Critical residues for binding are shown as lines colored by atom types. Hydrogen bonds are shown as dotted yellow lines with the distance between donor and acceptor atoms indicated. Atom type colour code: red for oxygen, blue for nitrogen, grey for carbon and yellow for sulphur atoms respectively. The HCR-13_pf_ residues that interact with the lead molecule are indicated by the arrow. The Maybridge database ID of the lead molecules are as follows: compound 1–3764; compound 2–764; compound 3–13196; compound 4–3705; compound 5–632; compound 6–7789; and compound 7–1598.

The structural scaffold of the lead molecules contains carboxylic acid functional group, mainly responsible for the hydrogen bond(s) formed with the HCR-13_pf_. The binding conformation of the lead molecules with the hydrogen bond interactions to the Hpx domain of MMP-13 are given in Figure 3. The short hydrogen bond distances, ranging from ∼1.5 to ∼2.4 Å, and the favourable binding G-scores (−9.22 to −7.55 kcal/mol) (Table 1) suggest strong enzyme-ligand interactions. These carboxylic acid containing lead molecules were found to exhibit hydrophilic contacts with 1PEX, mostly with the polar side chains of amino acids Arg344 and Arg346 of HCR-13_pf_. They also exhibited polar interaction with other functionally important amino acid residues that are not part of HCR-13, namely Arg300 and Lys347.

In accordance with Lipinski's rule of five, the Mobyle portal was used to evaluate the drug-likeness of the lead molecules by assessing their physicochemical properties. Their molecular weights were <500 daltons with <5 hydrogen bond donors, <10 hydrogen bond acceptors and a log p of <5 (Table S4); these properties are well within the acceptable range of the Lipinski rule for drug-like molecules. These compounds were further evaluated for their drug-like behaviour through analysis of pharmacokinetic parameters required for absorption, distribution, metabolism, excretion and toxicity (ADMET) by use of QikProp. For the seven lead compounds, the partition coefficient (QPlogPo/w) and water solubility (QPlogS), critical for estimation of absorption and distribution of drugs within the body, ranged between ∼ −0.1 to ∼2.3 and ∼ −4 to ∼ −0.05, cell permeability (QPP_Caco_), a key factor governing drug metabolism and its access to biological membranes, ranged from ∼26 to ∼276, while the bioavailability and toxicity were from ∼3.4 to ∼0.4. Overall, the percentage human oral absorption for the compounds ranged from ∼46 to ∼79%. All these pharmacokinetic parameters are within the acceptable range defined for human use (see Table 2 footnote), thereby indicating their potential as drug- like molecules.

**Table 2:** QikProp properties of the seven lead molecules, by use of Schrodinger 9.0.

Lead molecules a	QPlogPo/w b	QPlogS c	QPP_Caco_ d	QPlogHERG e	Percent human oral absorption f
3764	0.841	−1.388	70.704	0.219	47.742
764	2.239	−3.622	78.429	2.381	30.962
13196	2.239	−3.622	78.428	−3.382	73.962
3705	1.058	−1.740	44.646	−1.559	62.677
632	1.409	−1.558	276.206	−1.496	78.888
7789	−0.967	−0.051	26.073	0.351	47.633
1598	1.454	−1.630	249.077	−1.869	78.349

a Ligand IDs are of the Maybridge database.

b Predicted octanol/water partition co-efficient log p (acceptable range: −2.0 to 6.5).

c Predicted aqueous solubility; S in mol/L (acceptable range: −6.5 to 0.5).

d Predicted Caco-2 cell permeability in nm/s (acceptable range: <25 is poor and >500 is great).

e Predicted IC_50_ value for blockage of HERG K^+^ channels (acceptable range: below −5.0).

f Percentage of human oral absorption (<25% is poor and >80% is high).

As of May 2010, the number of MMP sequences in the NCBI Entrez protein public database almost doubled since our last data collection (May 2009). The May 2010 data contained a total of 94 MMP sequences, an increase of 44 since May 2009. Analysis of the 94 sequences revealed that the number of HCR-13 residues (completely conserved and unique to MMP-13) reduced significantly from 34 to only 10 (Gln309, Ala312, Lys318, His334, His337, Arg344, Asn352, Lys372, Ser378, and Glu373), whereas the HCR-13_pf_ reduced from 5 to 3 (Lys318, Arg344, and Lys372). This was expected because of our small initial sample size. Nonetheless, there was no change in the HCR-13_pf_ residues bound by our seven lead molecules, except for two (compounds 1 and 6). The putative functional residue Arg346 that interacts with both these compounds is no longer classified as an HCR-13, but the compounds still bind to one other HCR-13_pf_ residue (Table 1).

### Conclusion

The present work describes the identity of the putative functional residues characteristic to Hpx domain of MMP-13, and the identification of seven lead drug-like molecules binding to the HCR-13_pf_, with no observable cross-reactivity to MMP-1 and MMP-8. These molecules are potential selective inhibitors of MMP-13 that need to be experimentally validated, while the systematic cheminformatics-based drug design approach applied herein can be used for rational search of other public/commercial combinatorial libraries for more potent molecules, capable of selectively inhibiting the collagenolytic activity of MMP-13. Further, the backbone structural scaffold of these seven lead compounds could serve as building blocks in designing drug-like molecules in the treatment of OA, RA and other inflammatory disorders.

## Supporting Information

Table S1 Number of sequences of the various MMPs studied. These sequences were obtained from the NCBI Entrez protein database by use of PSI-BLAST search (as of May 2009).
(0.03 MB DOC)

Table S2 NCBI Entrez protein database accession and GI numbers of the 50 sequences analysed in this study.
(0.05 MB DOC)

Table S3 The 25 screened ligands that interact with at least one residue of the HCR-13_pf_ of MMP-13 (PDB ID: 1PEX) and their hydrogen bond interaction(s) to residues of MMP-1 (PDB ID: 1SU3) and -8 (PDB ID: 1BZS). 1SU3 structure contains both Hpx and catalytic domains, while 1BZS has only the catalytic domain. Rows shaded in grey are for the seven lead molecules.
(0.06 MB DOC)

Table S4 ADMET properties calculated using Mobyle portal for the seven lead molecules.
(0.04 MB DOC)
