# Supplementary material for: Cheminformatics-Based Drug Design Approach for Identification of Inhibitors Targeting the Characteristic Residues of MMP-13 Hemopexin Domain
Source: PLoS One. 2010 Aug 31;5(8):e12494. doi: 10.1371/journal.pone.0012494 (PMC2930869; doi:10.1371/journal.pone.0012494)
Supplement: Table S1 — Number of sequences of the various MMPs studied. These sequences were obtained from the NCBI Entrez protein database by use of PSI-BLAST search (as of May 2009). (0.03 MB DOC) [file pone.0012494.s001.doc]

**Table S1.** Number of sequences of the various MMPs studied. These sequences were obtained from the NCBI Entrez protein database by use of PSI-BLAST search (as of May 2009).

| MMP types | Number of sequences |
| --- | --- |
| MMP-1 | 14 |
| MMP-2 | 7 |
| MMP-3 | 2 |
| MMP-8 | 5 |
| MMP-10 | 2 |
| MMP-12 | 6 |
| MMP-13 | 8 |
| MMP-16 | 2 |
| MMP-24 | 1 |
| MMP-27 | 3 |
| Total | 50 |
