# Supplementary material for: Cheminformatics-Based Drug Design Approach for Identification of Inhibitors Targeting the Characteristic Residues of MMP-13 Hemopexin Domain
Source: PLoS One. 2010 Aug 31;5(8):e12494. doi: 10.1371/journal.pone.0012494 (PMC2930869; doi:10.1371/journal.pone.0012494)
Supplement: Table S2 — NCBI Entrez protein database accession and GI numbers of the 50 sequences analysed in this study. (0.05 MB DOC) [file pone.0012494.s002.doc]

**Table S2.** NCBI Entrez protein database accession and GI numbers of the 50 sequences analysed in this study.

| Accession Number | | GI Number |
| --- | --- | --- |
| AAP78939.1 | | 32306837 |
| EAW67034.1 | | 119587438 |
| BAF84900.1 | | 158257854 |
| AAH67523.1 | | 45768662 |
| BAG37740.1 | | 189067481 |
| BAD96468.1 | | 62897055 |
| NP_002418.1 | | 4505209 |
| 1PEX_A |  | 157833465 |
| 2JXY_A |  | 189095927 |
| AAB36943.1 | | 1688260 |
| 3BAO_A |  | 194709131 |
| AAA58658.1 | | 435970 |
| NP_002417.2 | | 73858572 |
| AAI43774.1 | | 219518916 |
| Q9H306.1 |  | 74725046 |
| NP_071405.2 | | 73808268 |
| AAQ89112.1 | | 37182623 |
| AAA36321.1 | | 188619 |
| NP_002413.1 | | 4505217 |
| NP_002416.1 | | 4505205 |
| BAD96321.1 | | 62896761 |
| BAG64444.1 | | 194382548 |
| BAF85348.1 | | 158258755 |
| AAZ38714.1 | | 71648770 |
| NP_002415.1 | | 4505221 |
| EAW67027.1 | | 119587431 |
| CAA28858.1 | | 38267 |
| BAD97001.1 | | 62898123 |
| AAA35699.1 | | 180665 |
| BAD96756.1 | | 62897633 |
| BAG62888.1 | | 194376258 |
| BAD96755.1 | | 62897631 |
| BAD96776.1| | | 62897673 |
| NP_002412.1 | | 4505215 |
| 1SU3_A |  | 58176727 |
| AAV38949.1 | | 54697154 |
| BAD96z754.1 | | 62897629 |
| NP_001139410 | | 225543094 |
| 2CLT_A |  | 114793614 |
| AAH13118.1 | | 15341877 |
| BAG35588.1 | | 189053422 |
| NP_001121363 | | 189217853 |
| 1CK7_A |  | 5822007 |
| BAG63035.1 | | 194376948 |
| 1GEN_A |  | 157831176 |
| NP_004521.1 | | 11342666 |
| AAA35701.1 | | 180671 |
| ABC17785.1 | | 83375894 |
| NP_005932.2 | | 13027802 |
| BAA23742.1 | | 2662306 |
|  | |  |
