# Supplementary material for: Cheminformatics-Based Drug Design Approach for Identification of Inhibitors Targeting the Characteristic Residues of MMP-13 Hemopexin Domain
Source: PLoS One. 2010 Aug 31;5(8):e12494. doi: 10.1371/journal.pone.0012494 (PMC2930869; doi:10.1371/journal.pone.0012494)
Supplement: Table S3 — The 25 screened ligands that interact with at least one residue of the HCR-13pf of MMP-13 (PDB ID: 1PEX) and their hydrogen bond interaction(s) to residues of MMP-1 (PDB ID: 1SU3) and -8 (PDB ID: 1BZS). 1SU3 structure contains both Hpx and catalytic domains, while 1BZS has only the catalytic domain. Rows shaded in grey are for the seven lead molecules. (0.06 MB DOC) [file pone.0012494.s003.doc]

**Table S3**. The 25 screened ligands that interact with at least one residue of the HCR-13_pf_ of MMP-13 (PDB ID: 1PEX) and their hydrogen bond interaction(s) to residues of MMP-1 (PDB ID: 1SU3) and -8 (PDB ID: 1BZS). 1SU3 structure contains both Hpx and catalytic domains, while 1BZS has only the catalytic domain. Rows shaded in grey are for the seven lead molecules.

| Ligand ID ^a^ | 1PEX  (Hpx domain) ^b^ | 1SU3 ^c^ | | 1BZS ^c^  (Catalytic domain) |
| --- | --- | --- | --- | --- |
|  |  | Catalytic domain | Hpx domain |  |
|  |  |  |  |  |
| 4261 | **ARG344,** ARG300, *ASN326* | LYS63, GLN66, ASP96 | LYS362, ARG405, ARG341 | **GLU198**, ALA161, LEU160 |
| 11798 | **ARG344**, **ARG346**, *ASN326* | GLN66, ASP96 | - | **GLU198**, ALA161, LEU160 |
| 3764 | **ARG344**, **ARG346,** LYS347, *ASN326* | LYS63, GLN66, ASP96 | - | ALA161, LEU160 |
| 9526 | **ARG344,** LYS347, *ASN326* | LYS63, GLN66, ASP96 | - | **GLU198**, TYR216, PRO217 |
| 764 | **ARG344,** ARG300 | GLN66, ASP96 | - | - |
| 12416 | **ARG344, ARG346**, LYS347, *ASN326* | LYS63, GLN66 | - | **GLU198**, HIS207, ALA161, LEU160 |
| 13575 | **ARG344, ARG346**, *ASN326* | ASP96 | - | **GLU198**, ALA161, LEU160, |
| 4199 | **ARG344, ARG346**, ARG327, *ASN326* | LYS63, GLN66, ASP96 | - | **GLU198**, PRO217 |
| 11397 | **ARG344,** ARG300 | LYS63, GLN66, ASP96 | - | **GLU198**, ALA161, LEU160 |
| 7889 | **ARG344, ARG346,** LYS347 | ASP96 | - | **GLU198**, PRO217 |
| 13196 | **ARG344,** ARG300 | GLN66 | LYS425 | HIS197, LEU160 |
| 3705 | **ARG344**, LYS347**,** *ASN326* | GLN66, ASP96 | - | ALA161 |
| 4413 | **ARG344, ARG346**, *ASN326* | LYS63, GLN66 | - | **GLU198,** LEU160 |
| 6885 | **ARG344**, *ASN326* | GLN66 | LYS362 | **GLU198**, ALA220 |
| 8756 | **ARG344, ARG346** | LYS63, ASP96 | - | **GLU198**, ALA161, LEU160 |
| 897 | **ARG344** | ASP96 | - | **GLU198** |
| 632 | **ARG344,** *ASN326* | LYS63, GLN66 | - | ALA161, PRO217 |
| 14575 | **ARG346,** LYS347 | LYS63, VAL97, ASP96 | - | **GLU198**, ALA161, LEU160 |
| 14196 | **ARG344,** ARG300, LYS347 | LYS63, ASP96 | - | **GLU198**, ALA161 |
| 3048 | **ARG344, ARG346,** LYS347, ARG327*, ASN326* | ARG91 | TYR360, ARG341, ARG405 | **GLU198** |
| 6607 | **ARG344,** ARG300 | LYS63, GLN66, ASP96 | LYS362, SER367, ARG405 | **GLU198**, LEU160 |
| 7832 | **ARG344,** ARG300, *ASN326* | GLN66 | - | **GLU198**, LEU160 |
| 1698 | **ARG344**, *ASN326* | LYS63, GLN66 | - | **GLU198** |
| 7789 | **ARG344, ARG346,** LYS347 | GLN66, ALA184, ASP96 | - | ALA220 |
| 1598 | **ARG344**, *ASN326* | LSY63, GLN66 | - | ALA161 |

^a^ Ligand IDs are of the Maybridge database.

^b^ The HCR-13 amino acids are in boldface; HCR-13_pf_ in boldface and underlined; functionally important residues of MMP-13 that are not part of HCR-13 are in non-boldface and underlined, while residues not functionally defined and not part of HCR-13 are in italics.

^c^ The information for the catalytic active sites of 1SU3 and 1BZS was obtained from PDBSum database. Active site residues of the catalytic domain are in boldface. None of the active site residues of the catalytic domain and the putative functional residues of Hpx of MMP-1 was bound by any of the 25 ligands.
