# Supplementary material for: Cheminformatics-Based Drug Design Approach for Identification of Inhibitors Targeting the Characteristic Residues of MMP-13 Hemopexin Domain
Source: PLoS One. 2010 Aug 31;5(8):e12494. doi: 10.1371/journal.pone.0012494 (PMC2930869; doi:10.1371/journal.pone.0012494)
Supplement: Table S4 — ADMET properties calculated using Mobyle portal for the seven lead molecules. (0.04 MB DOC) [file pone.0012494.s004.doc]

**Table S4**. ADMET properties calculated using Mobyle portal for the seven lead molecules.

| Parameters ^a^ | Ligand ID ^b^ | | | | | | | |
| --- | --- | --- | --- | --- | --- | --- | --- | --- |
|  | 3764 | 764 | 13196 |  | 3705 | 632 | 7789 | 1598 |
|  |  |  |  |  |  |  |  |  |
| MW | 182.1 | 185.1 | 170.1 |  | 185.1 | 179.1 | 172.1 | 179.1 |
| Drs | 0 | 0 | 0 |  | 1 | 0 | 1 | 0 |
| Ars | 4 | 5 | 4 |  | 5 | 4 | 5 | 4 |
| FB | 4 | 8 | 3 |  | 3 | 2 | 3 | 2 |
| RB | 8 | 4 | 9 |  | 7 | 12 | 7 | 12 |
| #R | 1 | 0 | 2 |  | 1 | 2 | 1 | 2 |
| RL | 6 | 0 | 5 |  | 6 | 6 | 5 | 6 |
| C | 8 | 7 | 5 |  | 6 | 9 | 7 | 9 |
| nC | 5 | 6 | 7 |  | 6 | 4 | 5 | 4 |
| C/nC | 0.63 | 0.86 | 1.40 |  | 1.00 | 0.44 | 0.71 | 0.44 |
| #Chrg | 2 | 3 | 1 |  | 1 | 1 | 1 | 1 |
| chrg | -2 | -3 | -1 |  | -1 | -1 | -1 | -1 |
| LogP | 0.53 | -2.36 | -1.17 |  | 0.14 | 0.82 | -1.59 | 0.73 |
| PSA | 73.94 | 110.91 | 82.86 |  | 108.28 | 55.43 | 77.51 | 55.43 |

^a^ Abbreviations: MW - Molecular weight, Drs - Hydrogen donors, Ars - Hydrogen acceptors, FB - Flexible bonds, RB - Rigid bonds, #R - Ring number, RL-Ring size, C - Number of atoms, nC - Atom number, C/nC - Ratio carbon/hetero, #Chrg - Charge number, chrg - Total charge, LogP -Partition of co-efficient, and PSA - Polar surface area.

^b^ Ligand IDs are of the Maybridge database.
